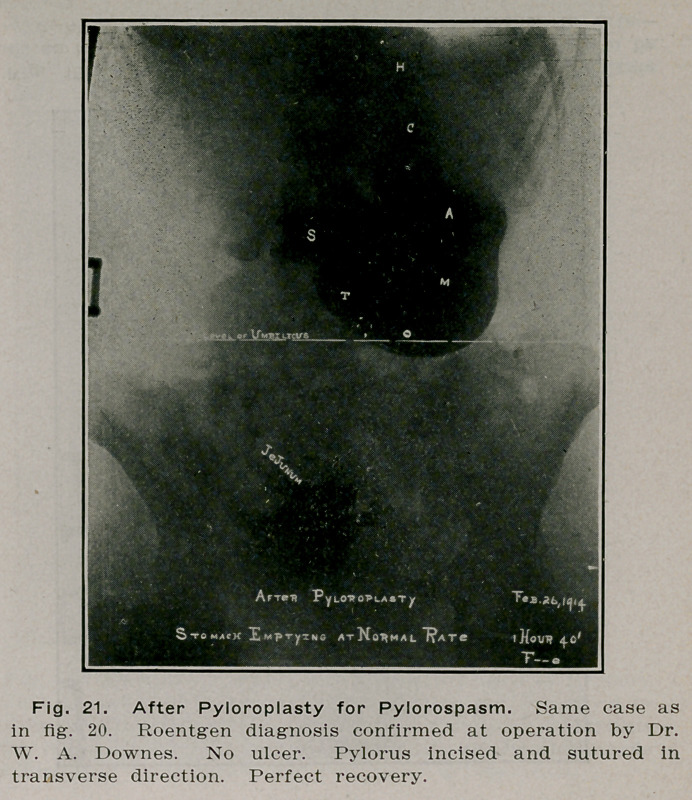# When to Operate in Chronic Conditions of the Stomach

**Published:** 1914-11

**Authors:** 


**When to Operate in Chronic Conditions of the Stomach.** By Walter A. Bastedo, M. D., and Leon Theodore Le Wald, M. D., New York. "American Medicine." Cuts furnished by courtesy of "American Medicine."

## Figures and Tables

**Fig. 2. f1:**
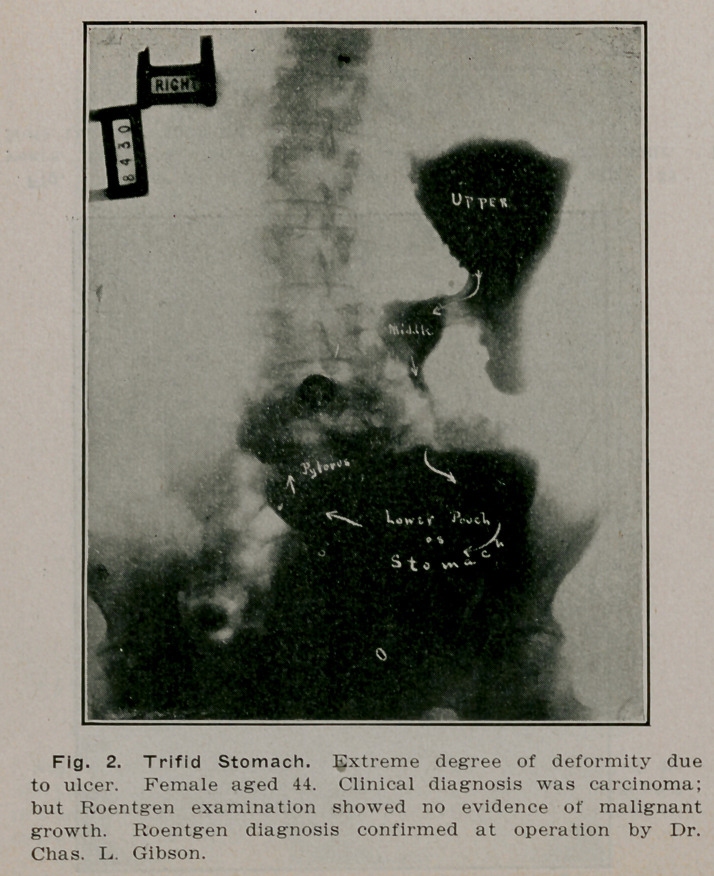


**Fig. 3. f2:**
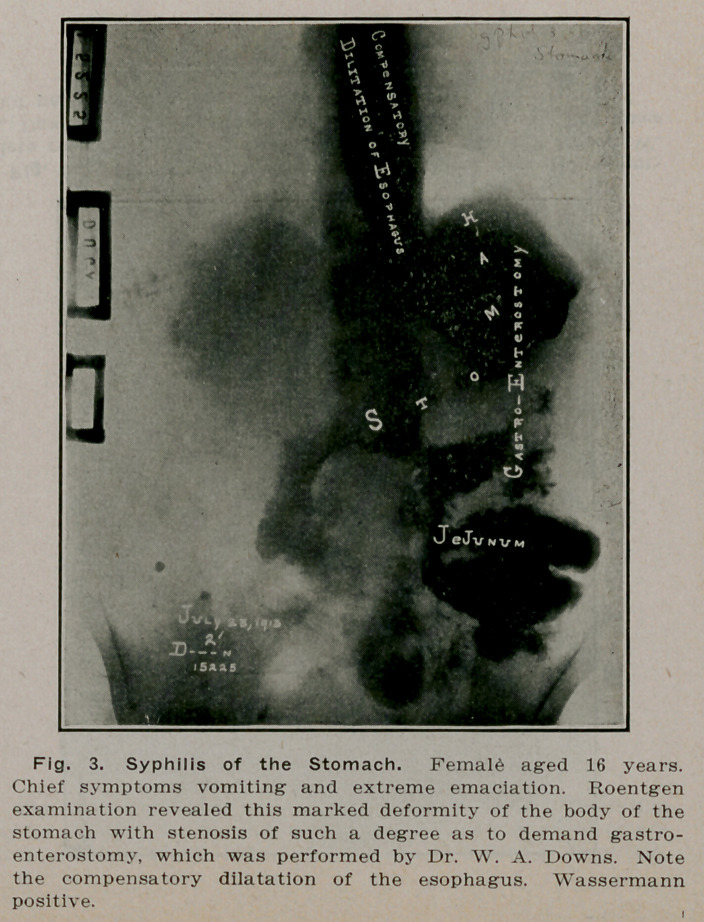


**Fig. 4. f3:**
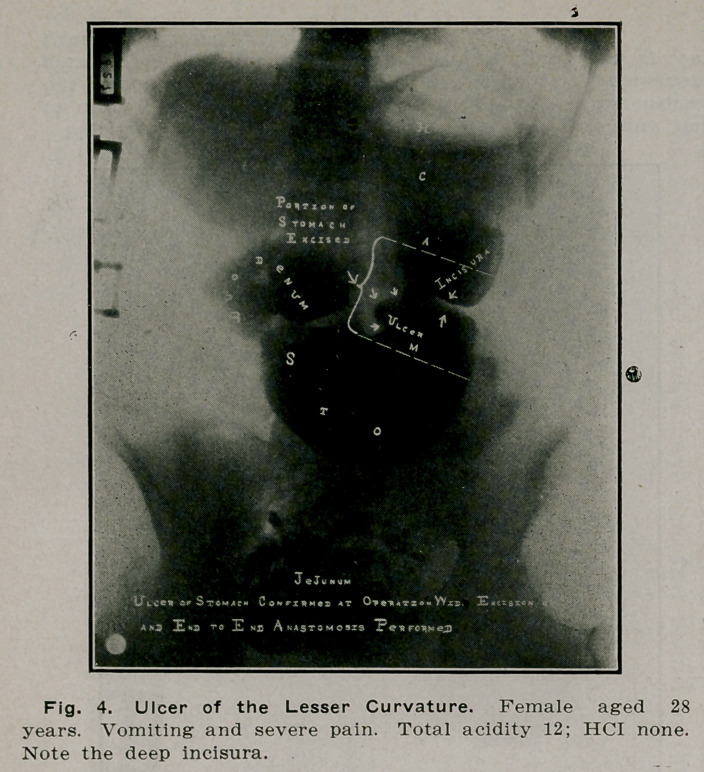


**Fig. 5. f4:**
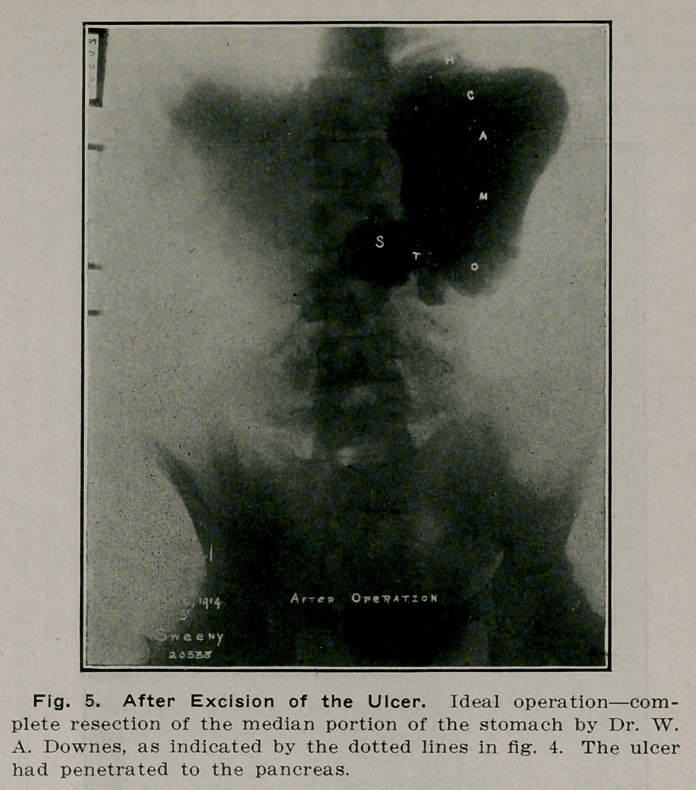


**Fig. 6. f5:**
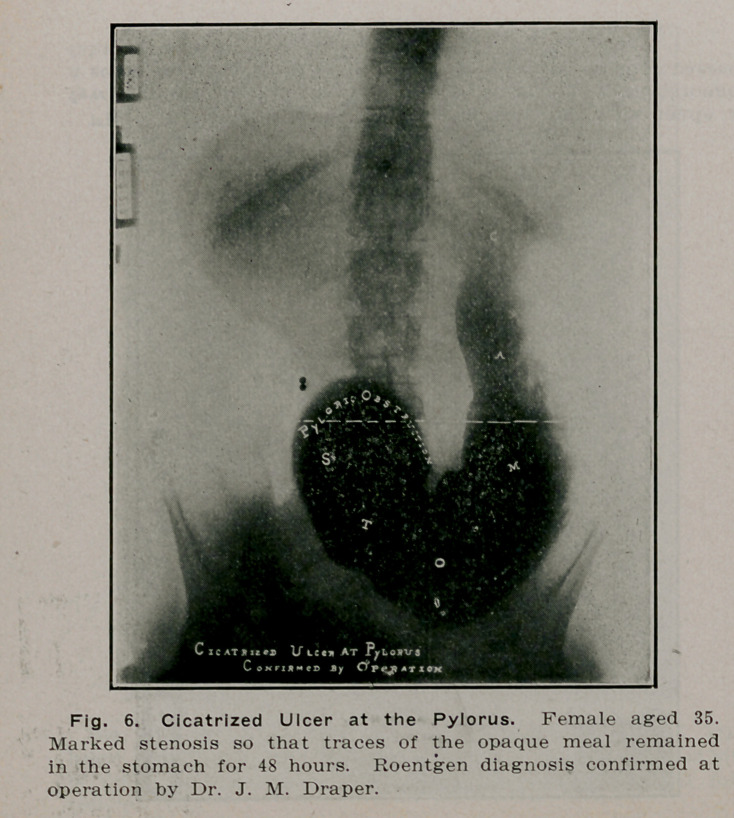


**Fig. 7. f6:**
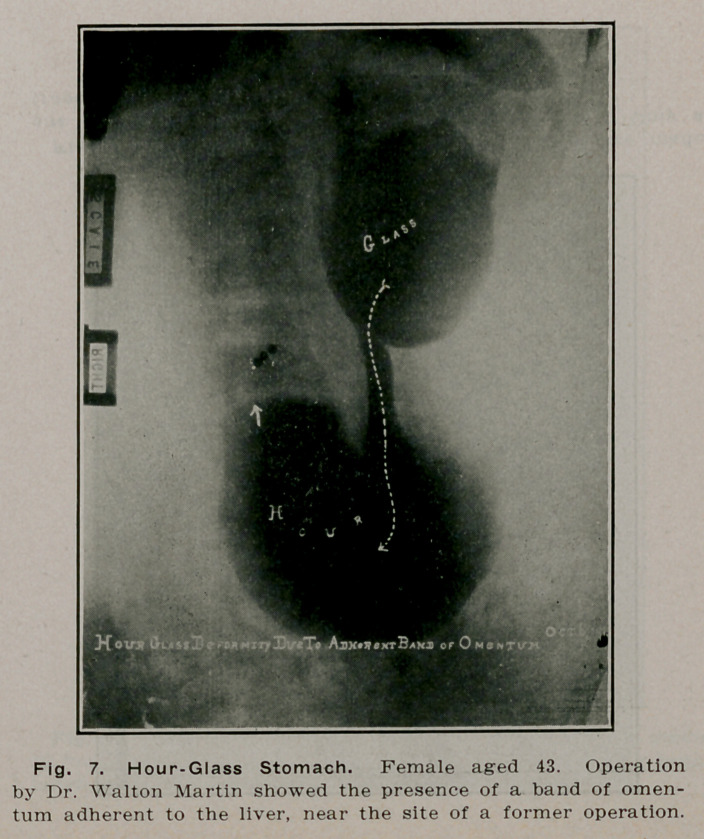


**Fig. 8. f7:**
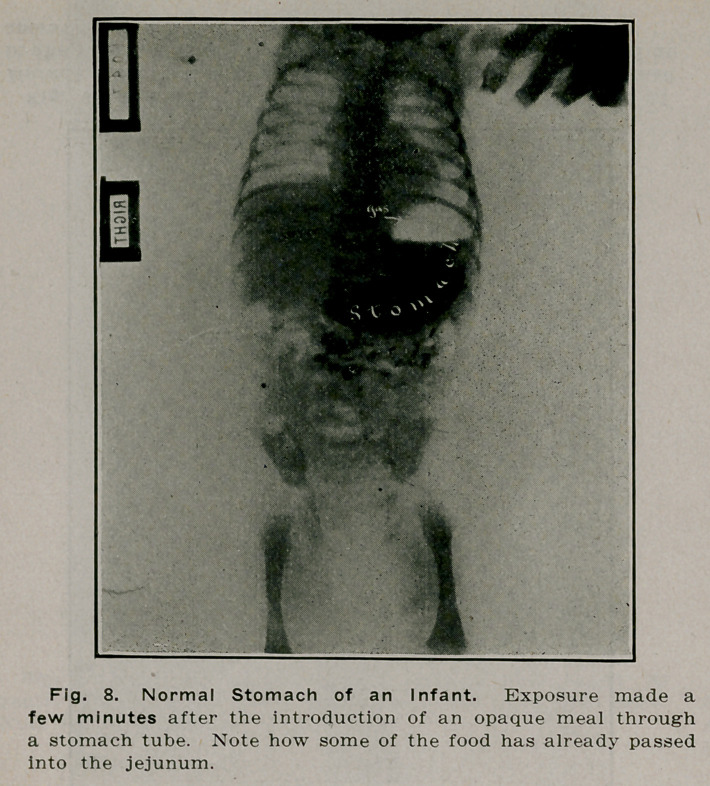


**Fig. 9. f8:**
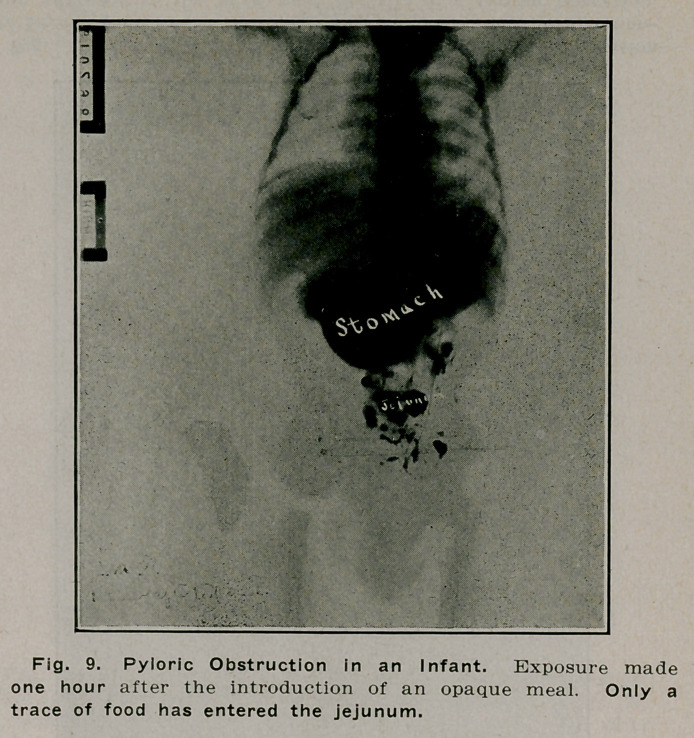


**Fig. 10. f9:**
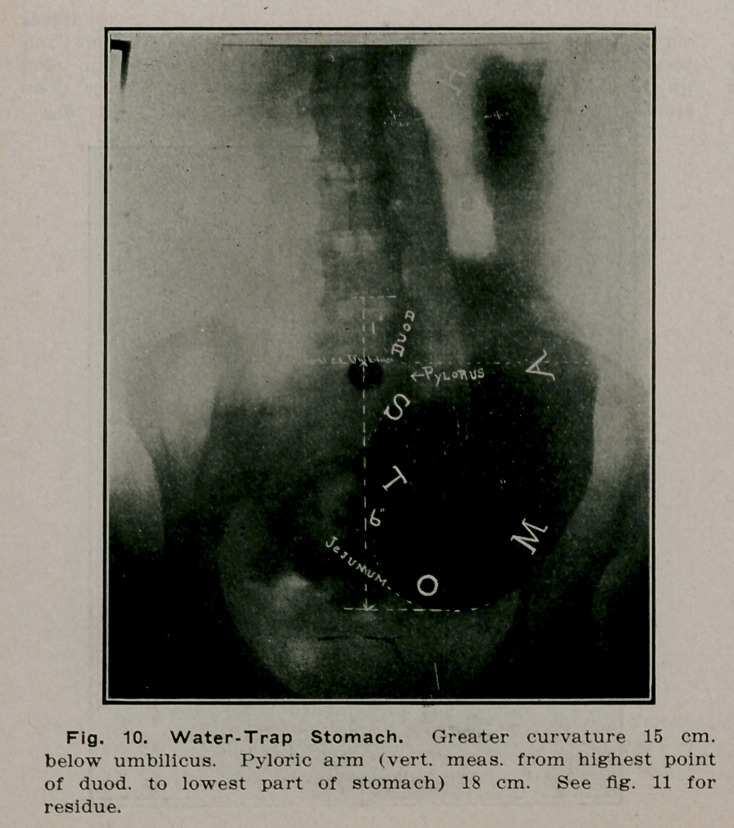


**Fig. 11. f10:**
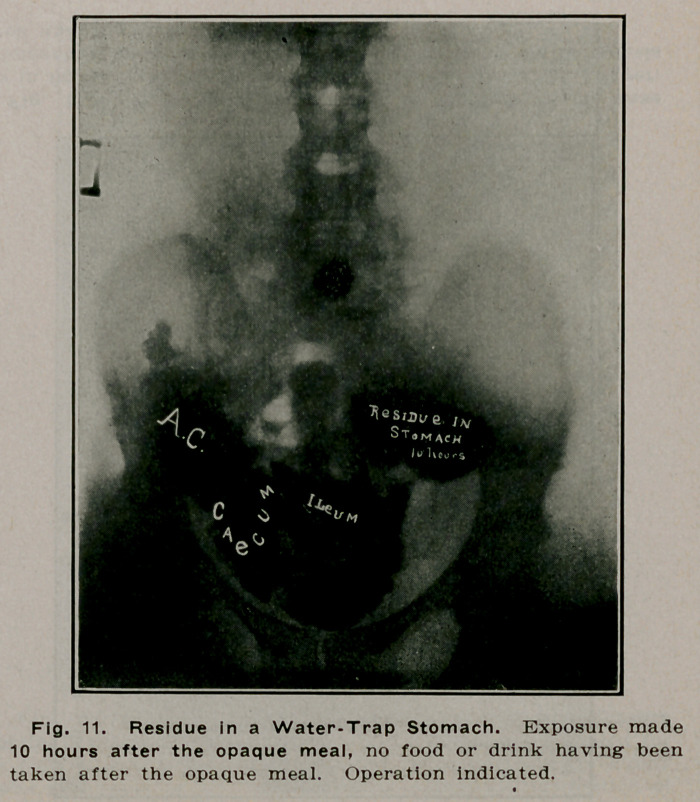


**Fig. 12. f11:**
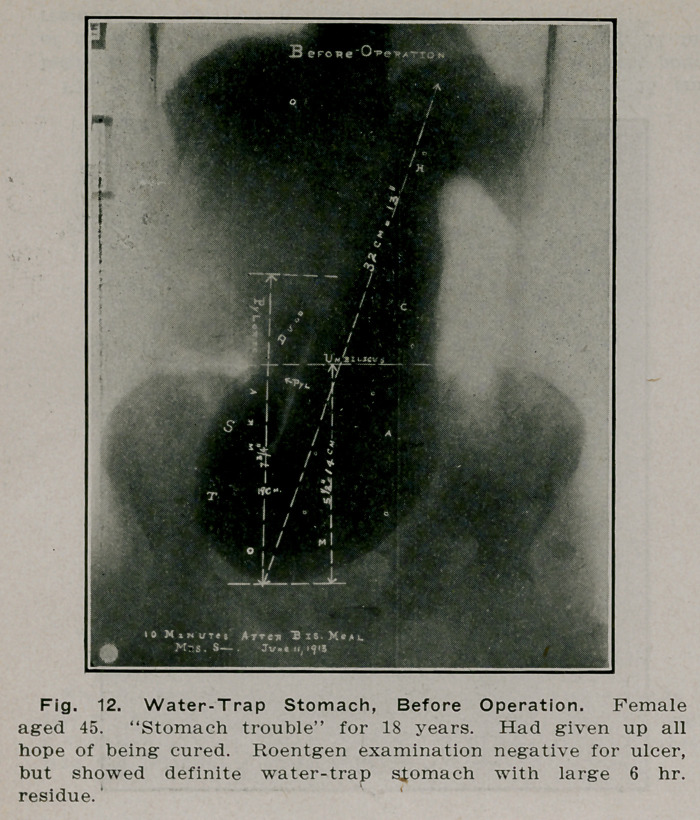


**Fig. 13. f12:**
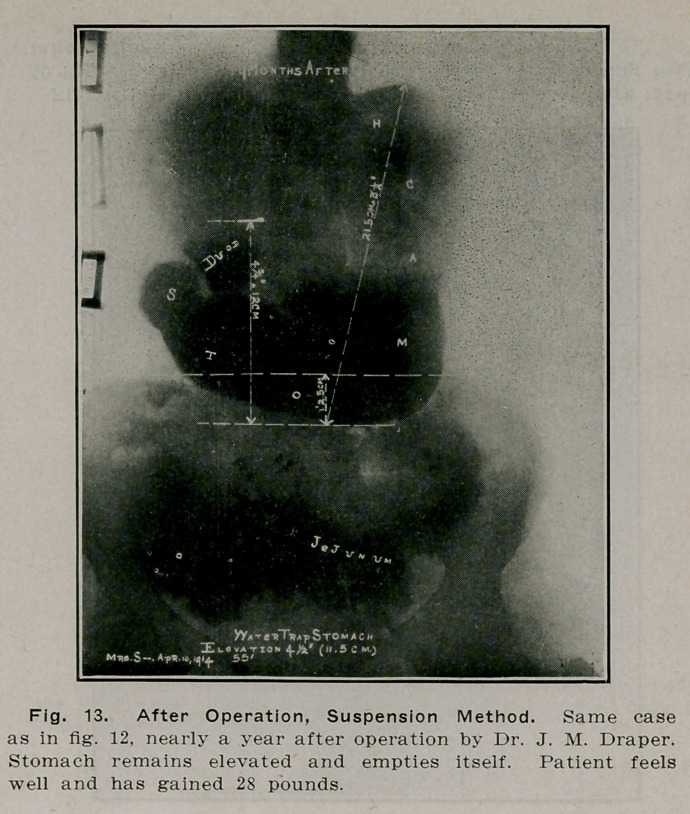


**Fig. 14. f13:**
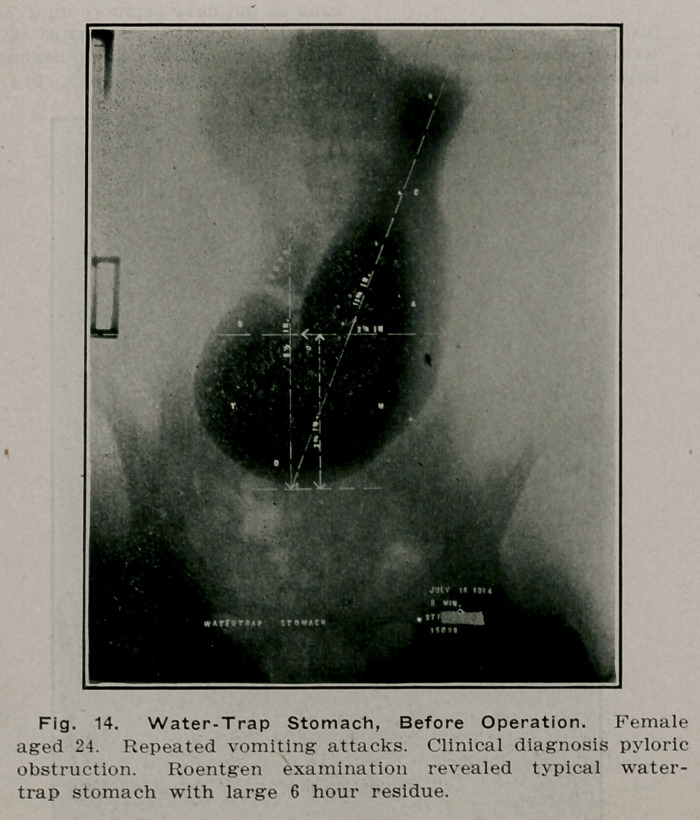


**Fig. 15. f14:**
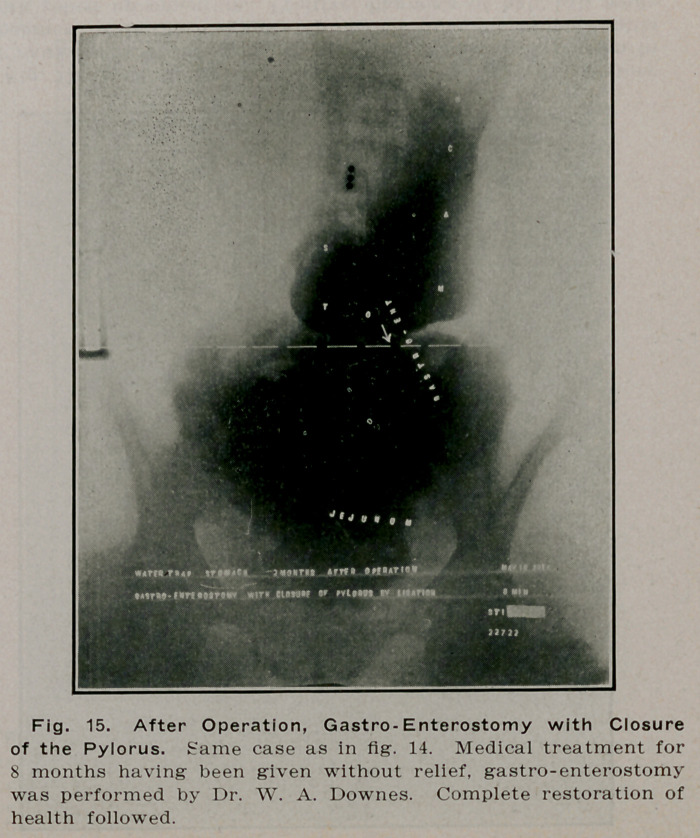


**Fig. 16. f15:**
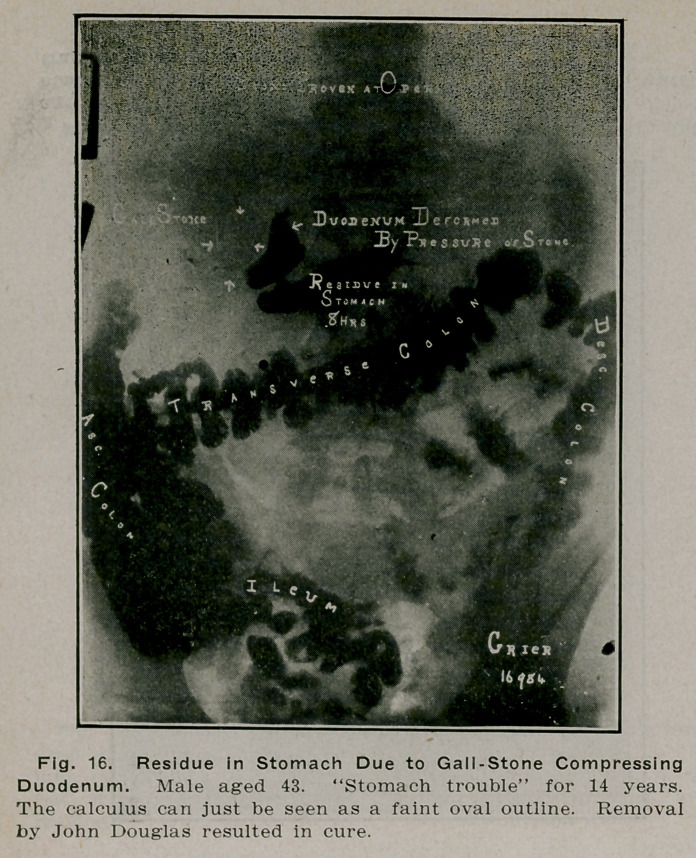


**Fig. 17. f16:**
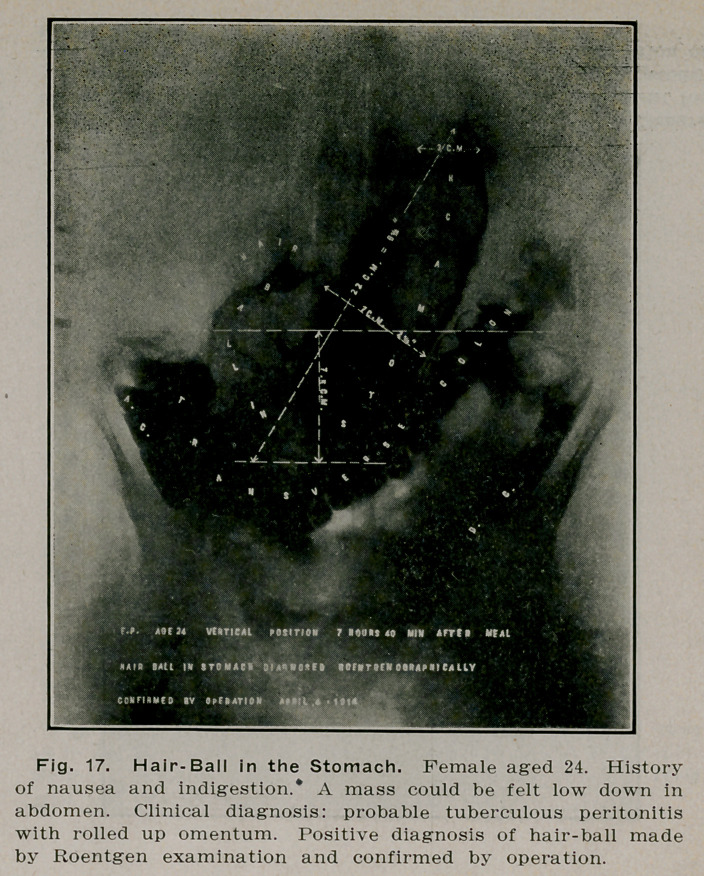


**Fig. 18. f17:**
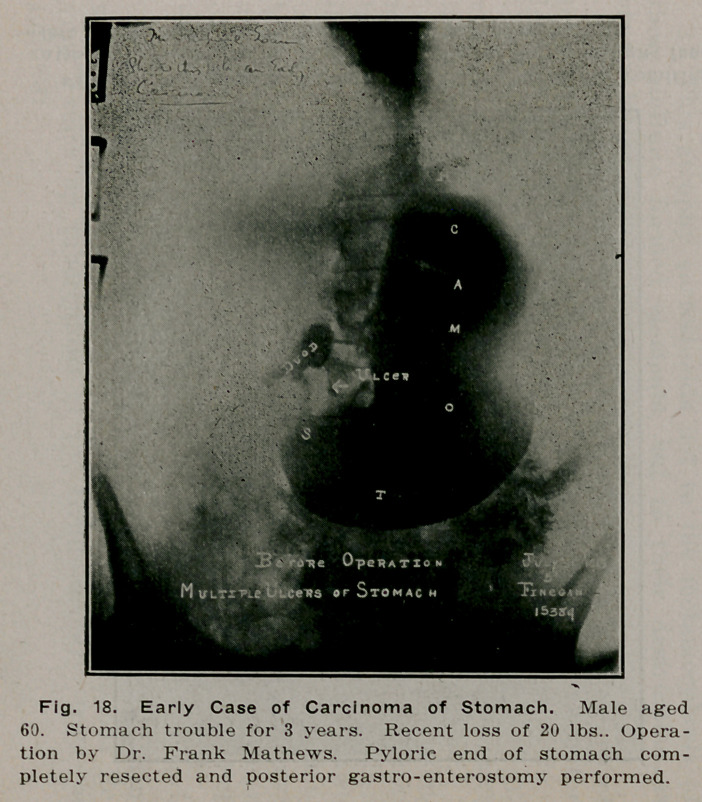


**Fig. 19. f18:**
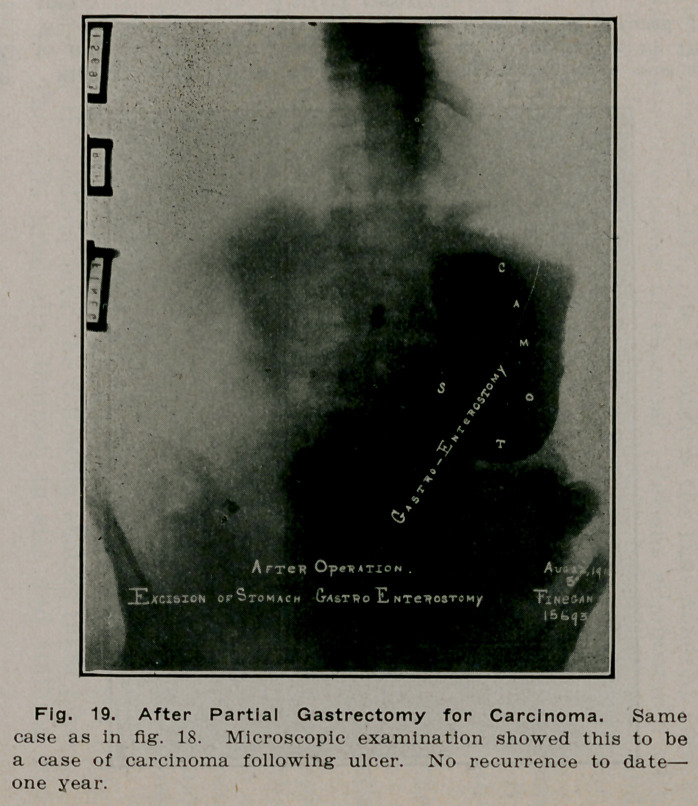


**Fig. 20. f19:**
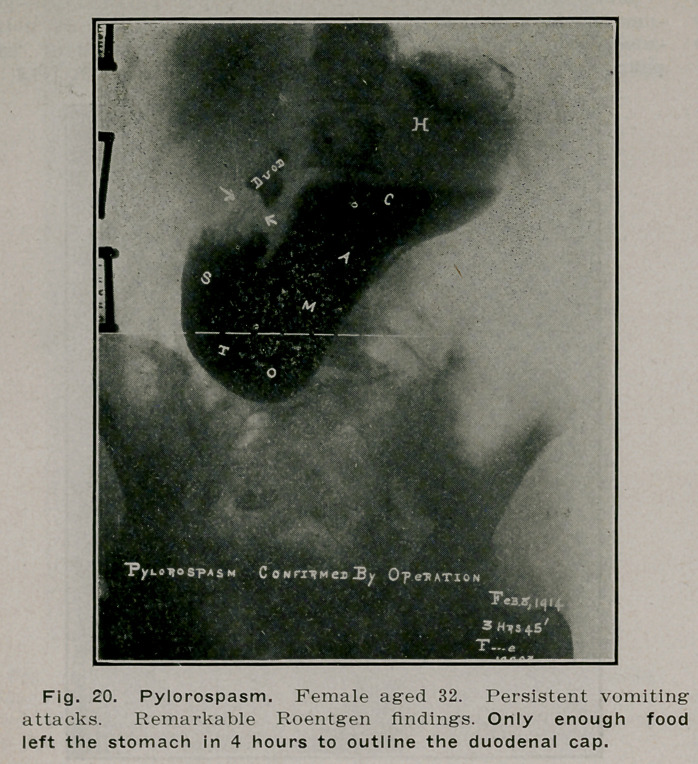


**Fig. 21. f20:**